# Iron oxide nanoparticles for neuronal cell applications: uptake study and magnetic manipulations

**DOI:** 10.1186/s12951-016-0190-0

**Published:** 2016-05-14

**Authors:** Michal Marcus, Moshe Karni, Koby Baranes, Itay Levy, Noa Alon, Shlomo Margel, Orit Shefi

**Affiliations:** Neuro-engineering lab, Faculty of Engineering, Bar Ilan University, Ramat Gan, Israel; Department of Chemistry, Bar Ilan University, Ramat Gan, Israel; Bar Ilan Institute of Nanotechnologies and Advanced Materials, Ramat Gan, Israel

**Keywords:** Magnetic nanoparticles, Magnetic field, Uptake, Neuronal cells, Cell positioning, Guidance, Neuronal regeneration

## Abstract

**Background:**

The ability to direct and manipulate neuronal cells has important potential in therapeutics and neural network studies. An emerging approach for remotely guiding cells is by incorporating magnetic nanoparticles (MNPs) into cells and transferring the cells into magnetic sensitive units. Recent developments offer exciting possibilities of magnetic manipulations of MNPs-loaded cells by external magnetic fields. In the present study, we evaluated and characterized uptake properties for optimal loading of cells by MNPs. We examined the interactions between MNPs of different cores and coatings, with primary neurons and neuron-like cells.

**Results:**

We found that uncoated-maghemite iron oxide nanoparticles maximally interact and penetrate into cells with no cytotoxic effect. We observed that the cellular uptake of the MNPs depends on the time of incubation and the concentration of nanoparticles in the medium. The morphology patterns of the neuronal cells were not affected by MNPs uptake and neurons remained electrically active. We theoretically modeled magnetic fluxes and demonstrated experimentally the response of MNP-loaded cells to the magnetic fields affecting cell motility. Furthermore, we successfully directed neurite growth orientation along regeneration.

**Conclusions:**

Applying mechanical forces via magnetic mediators is a useful approach for biomedical applications. We have examined several types of MNPs and studied the uptake behavior optimized for magnetic neuronal manipulations.

**Electronic supplementary material:**

The online version of this article (doi:10.1186/s12951-016-0190-0) contains supplementary material, which is available to authorized users.

## Background

The ability to manipulate and direct cells toward specific sites is of great importance in the field of biomedicine, with many potential implications in neurorepair therapies and in the development of bio-chip devices. Specifically, cell therapy for nervous system diseases and injuries includes the challenge of directing neural stem cells or engineered cells to the site of damage [1, 2]. Site restricted placement of the cells may enhance the efficiency of treatment and minimize systemic side effects [3]. Likewise, cell positioning is an issue in the creation of effective interfaces between neurons and devices [4–6]. For example, coupling and specific correspondence between neurons and electrical components is essential for accurate recordings and stimulations [7, 8].

A recent innovative and promising approach to achieve site specific targeting in vitro and in vivo is to form complexes of cells interacting with magnetic nanoparticles (MNPs) [9]. Due to their magnetic properties, magnetic nanoparticles (MNPs) experience force in inhomogeneous magnetic fields and hence can be manipulated through such fields [3, 10]. By incorporating MNPs within cells, cells can be directed to specific sites in response to external magnetic field gradients. In addition, MNPs can be functionalized with various biological molecules, i.e. proteins, nucleic acids, via chemical or physical conjugations, permitting them to specifically interact with the cells of interest within a region [10–12]. Interestingly, the conjugation of MNPs to various proteins, i.e. growth factors, has been shown to increase the proteins’ half-life and consequently enhance the effects on cells [13–15].

Recent studies have demonstrated the use of MNPs for the delivery of cells to key areas of specific organs. Yanai et al. magnetized rat mesenchymal stem cells using superparamagnetic iron oxide nanoparticles and magnetically targeted the cells to the upper hemisphere of a rodent retina [16]. Magnetic nanoparticles also have been used to guide endothelial progenitor cells through the blood stream to the brain cortex of mice using an external magnet [17]. Bone marrow stromal cells, labeled with magnetic beads, migrated through the cerebrospinal fluid to the desired site in the spinal cord in rats [18]. Magnetized stents were used to accumulate endothelial cells, loaded with MNPs, within blood vessels [19]. Magnetic neutrophils have been targeted to mice lungs under magnetic guidance following intravenous injection [20]. Jain et al. developed drug-loaded magnetic liposomes for magnetizing phagocytes (monocytes/neutrophils) and targeted to the brain under inflammatory condition [21, 22]. MNPs can be also used for cell delivery with the advantage of tracking under MRI [23].

Moreover, many efforts have been devoted to the development of magnetic devices for various cell manipulations [24]. Tseng et al. fabricated defined patterns of micromagnetic substrates in order to study cellular response to mechanical forces. By coalescing nanoparticles within cells, localized nanoparticle-mediated forces were applied approaching cellular tension [25]. Lee et al. developed a CMOS-microfluidic hybrid system for cell manipulations. An array of micro-electromagnets embedded in the CMOS chip control the motion of individual cells that are tagged with magnetic beads [26]. We have recently presented a method to locate cells on micro-scale pre-programmed magnetic pads that served as magnetic ‘hot spots’ [27]. Micro patterned magnetic arrays were also used for localizing nanoparticles at specific subcellular locations demonstrating the potential of stimulating specific activity within cells [28].

The emerging approach of using magnetic nanoparticles for cell positioning and manipulations, raises the need to evaluate and characterize the uptake properties conditions for optimal coalescing of nanoparticles and cells. In our study, we characterized and optimized cell uptake of MNPs to transfer neuronal cells into magnetic sensitive units. Due to the sensitivity of cells to MNP type, we characterized iron oxide nanoparticles with different cores and coatings, and examined their interaction with neurons and neuron-like cells. We studied uptake kinetics and the effect on cell viability. We examined whether the growth, morphology and electrical activity patterns of neuronal cells are affected by MNPs uptake. Finally, we demonstrated the response of MNP-loaded cells to controlled external magnetic fields and have shown effects on cell motility and pattern of growth.

## Methods

### Cells and cell culture

Rat pheochromocytoma PC12 cells (ATCC) were grown in suspension in the RPMI medium supplemented with 10 % horse serum (HS), 5 % fetal bovine serum (FBS), 1 % l-glutamine, 1 % penicillin–streptomycin and 0.2 % amphotericin, in a humidified incubator at 37 °C containing 5 % CO_2_ (medium and supplements were purchased from Biological Industries, Israel). To induce differentiation, cells were seeded on plates coated with collagen type l and incubated for 24 h in serum reduced media (1 % HS). Murine β-NGF (Peprotech, Israel) was then added to the medium. Every two days, cells were rinsed with PBS, and fresh medium and NGF were added to the cells. Human neuroblastoma SH-SY5Y cells (ATCC) were grown in Dulbecco’s modified Eagle’s medium supplemented with 10 % FBS, 1 % l-glutamine, 1 % penicillin–streptomycin and 0.2 % amphotericin. Leech neurons were isolated from the central nervous system of adult medicinal leeches *Hirudo medicinalis* as described in detail in Baranes et al. [29, 30].

PC12 cells were used for viability and uptake studies, morphology analysis and magnetic positioning experiments. SHSY-5Y cells were used as a complementary cell line to examine MNPs uptake by human cells. The primary leech neurons were used as a model for examining electrical activity and magnetic guidance of neurites via MNPs interactions at the single cell level.

### Magnetic nanoparticles

Four types of MNPs were used: (i) Maghemite (γ-Fe_2_O_3_) fluorinated magnetic nanoparticles synthesized by nucleation, followed by controlled growth of γ-Fe_2_O_3_ thin films onto gelatin RITC-iron oxide nuclei (RITC, Rhodamine Isothiocyanate) according to the description in previous publication [31]. (ii–iv) Magnetite (Fe_3_0_4_) core particles with different coatings (Chemicell, Berlin, Germany). We studied nano-screenMAG–UC/C (uncoated, cationic), nano-screenMAG-D (coated with starch) and nano-screenMAG-DXS (coated with dextran sulfate) particles. The nano-screenMAG particles consist of a magnetite core surrounded by a lipophilic fluorescent dye covered by a hydrophilic matrix (starch or dextran). The nanoparticles have a red fluorescence (excitation: 578; emission: 613) (Table 1).

**Table 1:** Summary of magnetic nanoparticle core and coating properties

Particle type	Hydrodynamic diameter (nm)	Dry diameter (nm)	Charge	Coating	Functional group
Uncoated-magnetite MNPs	100	10	Cationic	No coating	–
Starch-magnetite MNPs	100	10	Neutral	Starch	Hydroxyl groups
Dextran-magnetite MNPs	100	10	Neutral	Dextran	Sodium sulfate
Uncoated-maghemite MNPs	100	20	Anionic	No coating	Carboxyl and amine groups

### Flow cytometry analyses of nanoparticles uptake

To study the effect of incubation time on nanoparticle uptake, PC12 cells were incubated with MNPs for 1, 2, 3, 24, 48 and 72 h. In a separate experiment, to study the effect of MNPs concentration on cellular uptake, PC12 cells were incubated with MNPs at different concentrations, ranging from 0.01 to 0.5 mg/ml. Cells were then washed twice with fresh medium and collected in the dark. Fluorescence intensity in cells was measured by flow cytometry (FACS, Beckman Coulter Inc., CA, USA) with laser excitation at 488 nm and emission filtered at 600 nm, with 30 nm band width.

### Cell viability assay

The XTT assay was used to examine the cytotoxicity of the iron oxide nanoparticles. PC12 cells were seeded on 96-well plates. After 24 and 72 h of MNPs exposure, XTT reaction solution (Biological Industries, Israel) was added to the medium and incubated for 5 h at 37 °C. Absorbance was measured at 450 nm (630 nm background) using a spectrophotometer (BioTek Synergy4, Vermont USA).

### Imaging and morphometric analysis

Confocal microscopy imaging was performed using a Leica TCS SP5 microscope with an Acousto-Optical Beam Splitter. A light microscope (Leica DMIL LED) was used to acquire phase images of cultured cells and networks for image processing analysis. Morphometric parameters included neurite lengths, number of branching points and number of neurites originating from cell soma. We used NeuronJ, an ImageJ plugin (US National Institutes of Health, Bethesda, MD, USA), which enables semi-automatic tracing of neurites and length measurements [32]. Three batches of experiments were conducted. For each experiment, morphological parameters and statistics were measured for a total of 750 cells–125 cells per condition (control and MNPs treated) and per day (days one, three and five).

### Immunofluorescence staining

PC12 cells were fixed with 4 % paraformaldehyde for 15 min at room temperature, washed with PBS and permeabilized with 0.5 % Triton X-100 in PBS (PBT) for 10 min. Cells were then incubated in a blocking solution (containing 1 % bovine serum albumin (BSA) and 1 % normal goat serum (NGS) in 0.25 % PBT for 45 min. Next, cells were incubated with a rabbit antibody to α-tubulin (Santa Cruz Biotechnology, Inc., Santa Cruz, CA) overnight at 4 °C. The cells were rinsed with PBS and incubated for 45 min at room temperature with Cy2-conjugated AffiniPure Donkey Anti-rabbit secondary antibody (Jackson ImmunoResearch Laboratories, Inc., West Grove, PA). After incubation, cells were rinsed with PBS and mounted in an aqueous mounting medium.

### Electrophysiological measurements

Microelectrodes for intracellular recordings were made with borosilicate glass of 1 mm exterior diameter and 0.75 mm internal diameter pulled in a P97 puller (Sutter instruments) to create a tip diameter of 0.7–0.9 μm. The microelectrodes had resistances of 18–23 MΩ when filled with 3 M potassium acetate. We used a standard single-electrode current-clamp intracellular recording technique to monitor spike activity in leech neuronal culture. Signals are amplified (molecular devices multi clamp 700B) filtered and digitized by an analog-to-digital board Digidata 1400A (Axon instruments). Data were stored on a PC using pClamp 10.3 software (molecular devices).

### Magnetic tip preparation and magnetic field simulation

For magnetic cell positioning we designed magnetic tips on top of cylindrical magnets. The cylindrical magnets used and modeled (Metal Suppliers Online LLC, Hampstead, NH, USA) are axially magnetized, made of NdFeB N50, coated with nickel cooper nickel, with 18 mm in diameter and length of 18 mm. (Additional file 1: Figure S1.A). The tips were made from Hiperco 50A to ASTM A801 type 1, shaped as truncated cone with 18 mm base and 0.5 mm tip with total height of 18 mm. The cone-shaped and cylindrical magnets were placed in a suited plastic holder (Additional file 1: Figure S1.B). In experiments using two magnetic tips, each tip was formed of a cone-shaped magnet placed above two cylindrical magnets, with a controlled angle between the two magnetic tips (Additional file 1: Figure S1.C).

The magnetic flux density resulting from the different geometries was calculated by means of numerical field calculations using the software Comsol Multiphysics 4.3b (Comsol Multiphysics GmbH, Goettingen, Germany) for a stationary magnetic fields without current. A 3D model that was programmed in SolidWorks (Dassault Systèmes SOLIDWORKS, Waltham, Massachusetts, USA) was imported via the LiveLink™ for SOLIDWORKS Module of Comsol Multiphysics. A 3D simulation model was developed in the magnetic fields, no currents (mfnc) module in a stationary state, taking into consideration the thickness of plastic culture dish. The relevant Maxwell equations were solved for the imported 3D model and a finer physics-controlled mesh.

A digital Gauss-meter (Scientific Equipment Roorkee, DGM-204) was used to measure the magnetic field induced by the two setups of magnet tips.

### Statistical analysis

Error bars represent standard errors. All experiments were performed in triplicates and compared with the control using the *t* test. A *p* value of 0.05 was considered statistically significant.

## Results and discussion

### Magnetizing cells and effects on cell viability

We studied cell interactions with iron oxide nanoparticles with magnetite and maghemite cores, uncoated and coated, of the same hydrodynamic diameter of 100 nm (commercial and synthesized). Coatings included starch and dextran polymers that are expected to improve cellular uptake of the MNPs. Detailed description of studied MNPs, is summarized in the methods section. We examined four types of MNPs, which are labeled by their coating and core: uncoated-magnetite, starch-magnetite, dextran-magnetite and uncoated-maghemite MNPs.

**Fig. 1:**
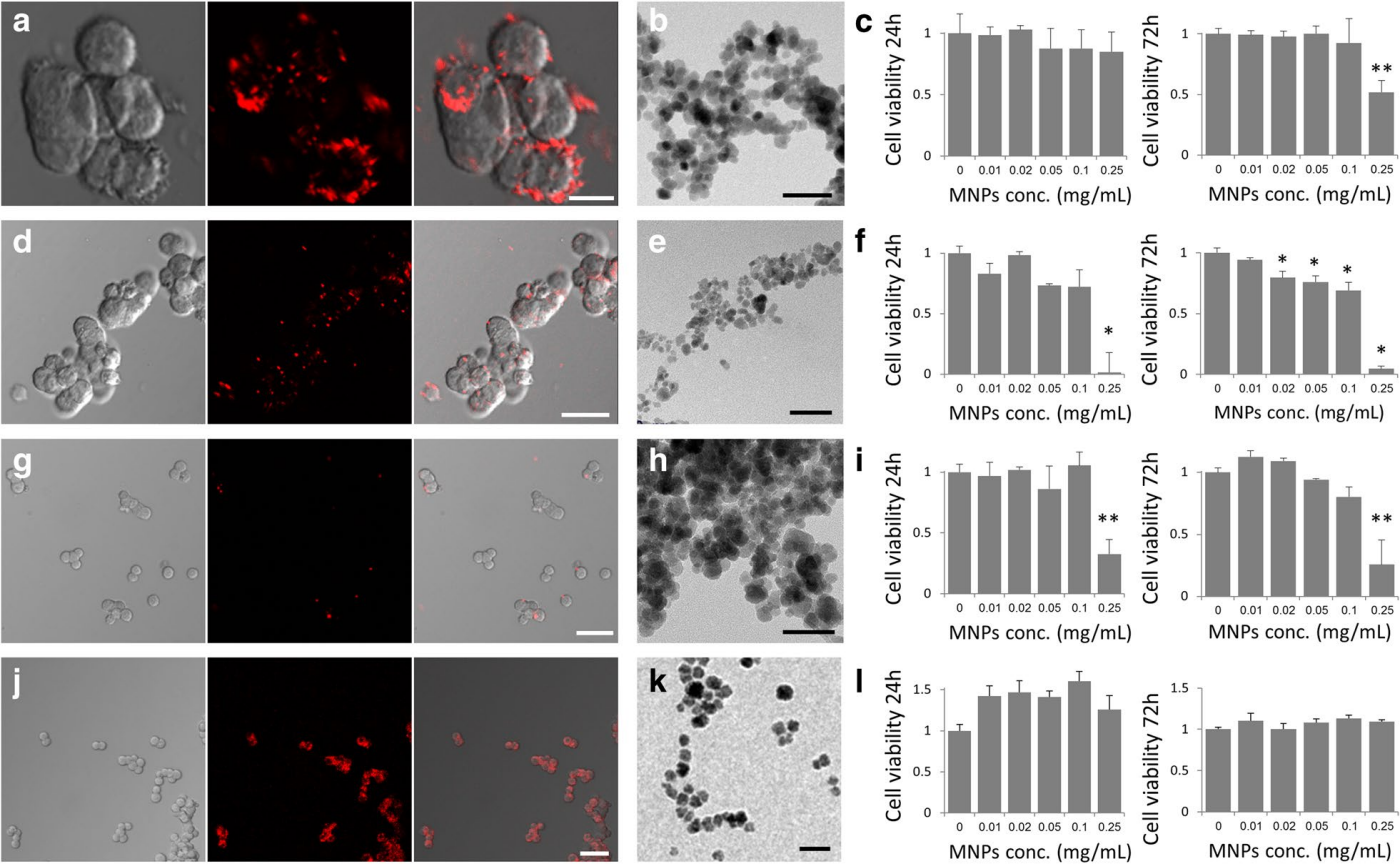
Characterization of the interactions of magnetic nanoparticles with PC12 cells: **a**–**c** uncoated-magnetite MNPs, **d–f** starch-magnetite MNPs, **g**–**i** dextran-magnetite MNPs, **j**–**l** uncoated-maghemite MNPs. *Left panel*: Confocal images of PC12 cells incubated with MNPs.* Scale bar* = 10, 25, 50 and 50 µm, respectively. *Middle panel*: TEM images of particles.* Scale bar* = 50 nm. *Right panel*: Cytotoxicity assay of cells incubated with increasing concentrations of MNPs after 24 and 72 h of incubation (*n* = 3). T test, *p < 0.05 and **p < 0.01

Figure 1 presents PC12 cells incubated with the four types of MNPs. It can be seen that MNPs with different characteristics interact with the cells in a different manner. Cells were incubated with MNPs for 24 h, washed twice and observed by confocal microscopy. Fluorescent confocal images show that uncoated-magnetite particles decorated the cells on the outer membrane and did not penetrate into the cells. Red fluorescence can clearly be seen constricted to cell membrane (Fig. 1a). Starch-magnetite particles bound to the outer membrane non-homogeneously as aggregates (Fig. 1d). The dextran-magnetite particles show no correlation with the cells. It seems that these particles were washed out and had no interaction with the cells (Fig. 1g). Figure 1j demonstrates that the uncoated-maghemite MNPs penetrated the PC12 cells. High fluorescence levels were detected within the cells. Uptake outcome for the examine MNPs is different, although the MNPs present same hydrodynamic diameter, within the optimal range for uptake of non-phagocytic cells [33–35].

To investigate the cytotoxicity of the MNPs, XTT cell viability assay was performed in time-dependent manner. PC12 cells were incubated with the different types of MNPs at concentrations ranging from 0.01–0.25 mg/ml. Assay results were normalized to cells with no particles. Uncoated-magnetite MNPs showed no cytotoxic effect up to 0.1 mg/ml (Fig. 1c). More than 90 % of cells remained viable. However, when incubated at a high concentration of 0.25 mg/ml, only 51 % of the PC12 cells remained viable after 72 h. Starch-magnetite MNPs showed a slight decrease in cell viability after 72 h as MNPs concentration increased (80 and 70 % viability at 0.02 and 0.1 mg/ml, respectively) (Fig. 1f). At a concentration of 0.25 mg/ml, MNPs were toxic to PC12 cells. After already 24 h no cells remained viable. Dextran-magnetite MNPs showed the same trend: cell viability decreased at a MNP concentration of 0.25 mg/ml (Fig. 1i). XTT assay of the uncoated-maghemite MNPs showed that increasing MNPs doses did not affect cell viability (Fig. 1l). No significant difference in cell viability was observed also after 5 days of incubation, indicating that these MNPs have no cytotoxic effect on PC12 cells (Additional file 2: Figure S2).

It has been previously reported that high dosages of iron oxide MNPs are toxic to PC12 cells [36, 37]. Our results show that the magnetite MNPs we examined are indeed toxic at high concentrations (>0.1 mg/ml). However, the uncoated-maghemite MNPs show no toxicity, enabling incubation of MNPs at high concentrations. Figure 1l presents the effect of up to 0.25 mg/ml. Non-toxic effect was measured up to 0.6 mg/ml (Additional file 2: Figure S2).

Our study of PC12 cells viability demonstrates differences in toxicity response clearly. As described previously, the toxicity of iron oxide particles varies between particle and cell type and depends on many factors, i.e. particle coating, level of aggregation, stability [35]. Numerous studies examining iron oxide particles imply that the toxicity is related to the particles’ coating characteristics and experimental conditions with no evidence to toxic effects of the magnetic core (maghemite or magnetite) [36, 38].

For further characterization of the MNPs we performed TEM imaging. It can be seen that uncoated-magnetite, starch-magnetite and dextran-magnetite MNPs show an average dry diameter of 10 nm (Fig. 1b, e, h). The uncoated-maghemite MNPs demonstrate a diameter of 23.0 ± 2.1 nm (Fig. 1k). The TEM images show that the magnetite MNPs tend to aggregate where the uncoated-maghemite MNPs remain finely dispersed. This may explain the penetration dynamics of the studied MNPs. Although the dry diameter of the uncoated-maghemite MNPs is larger, the avoidance of aggregation improve cells’ uptake.

These results present the sensitivity of cells to the type of MNPs presenting the need of optimization the specific MNPs to the desired application. Taking into consideration the uptake and viability assays, we specifically suggest the maghemite MNPs that are best tolerated by cells as the magnetic mediators suitable for magnetic manipulations.

### Cellular uptake study

**Fig. 2:**
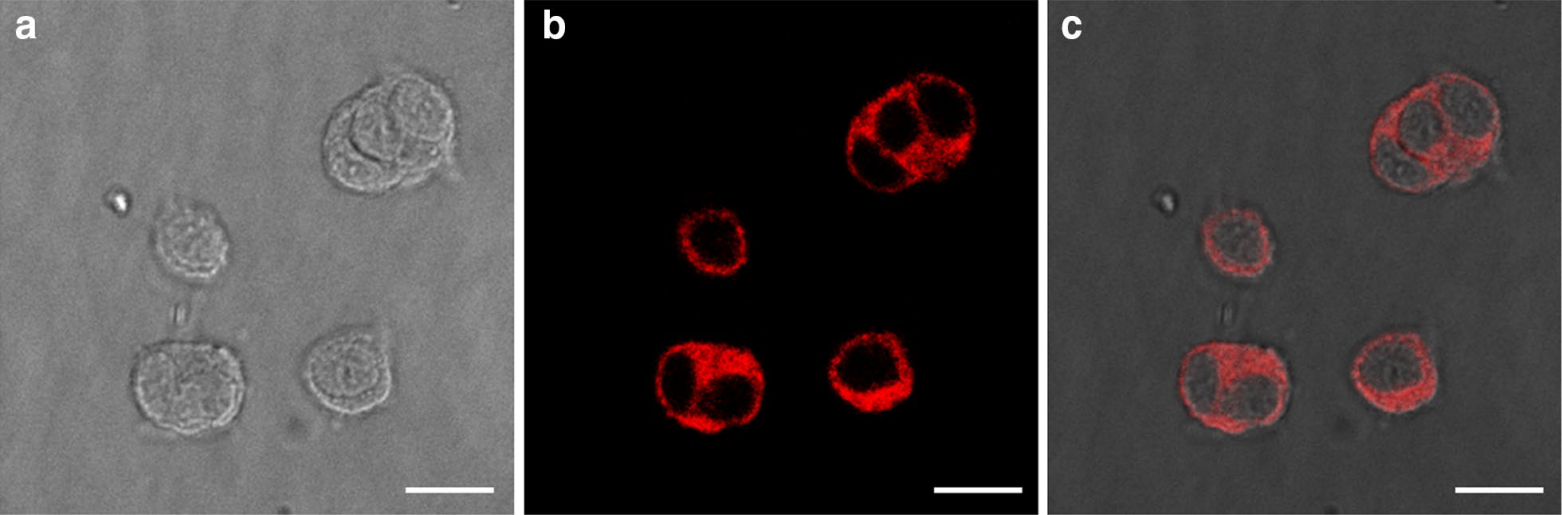
Confocal microscopy images of PC12 cells at a single focal plane after 24 h of incubation with the fluorescent uncoated-maghemite MNPs. MNPs labeled with rhodamine (*red*) enter the cells. **a** Phase contrast image. **b** Fluorescent image. **c** Merged image.* Scale bar* = 10 µm

**Fig. 3:**
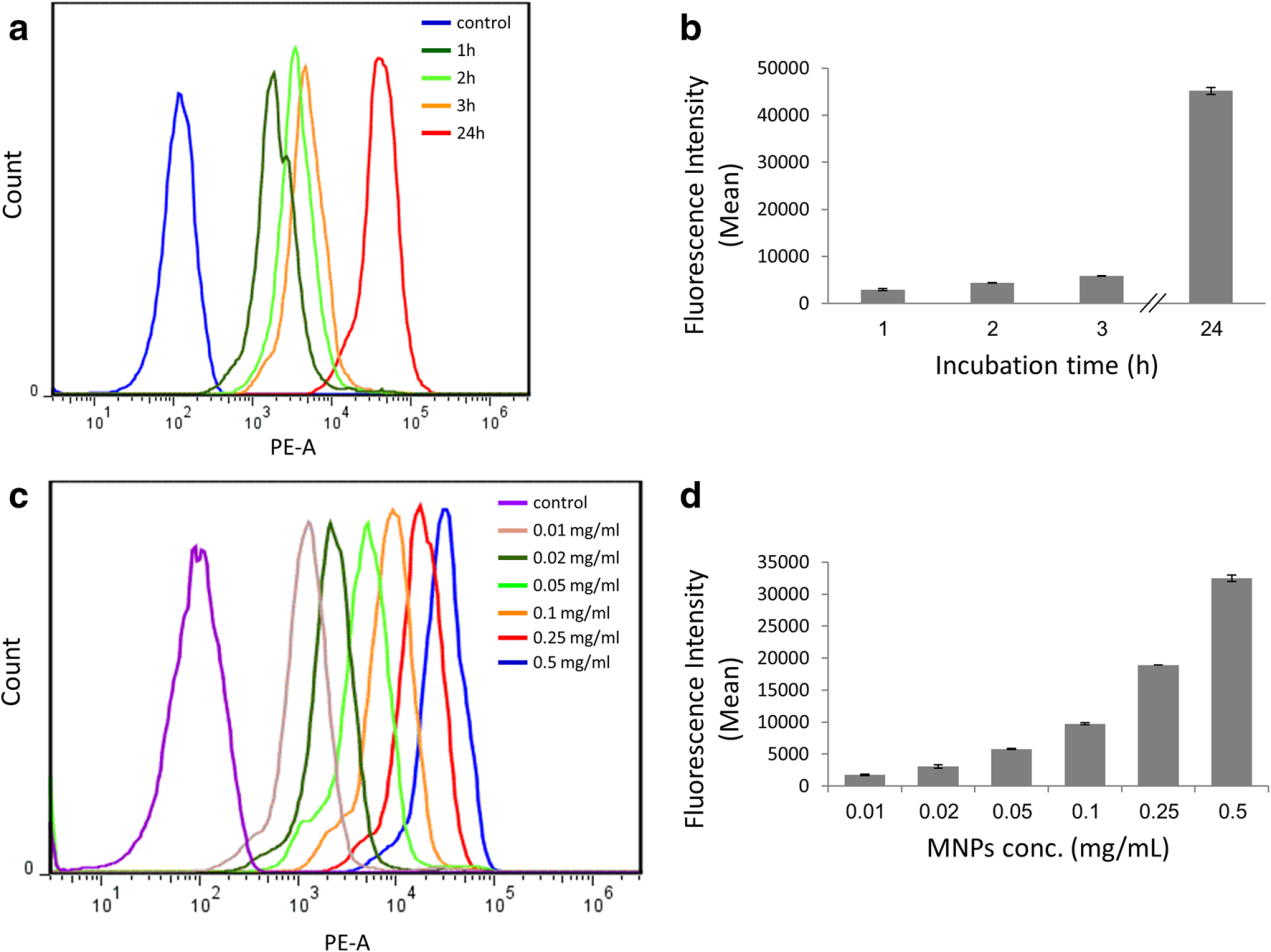
Cellular uptake of MNPs by PC12 cells; **a** Fluorescence intensity measurements from FACS of PC12 cells incubated with MNPs for 1, 2, 3 and 24 h. **c** Fluorescence intensity measurements from FACS of PC12 cells incubated with MNPs, ranging from 0.01 mg/ml to 0.5 mg/ml, for 24 h. **b**, **d** Average of fluorescence intensity normalized to control upon incubation of cells with MNPs. Reported values are an average of measurements (*n* = 3) of approximately 10,000 cells in each triplicate tested sample

We further studied the uptake of the maghemite nanoparticles by cells. Fluorescent confocal microscopy images of PC12 cells at a single focal plane verify the internalization of the MNPs into the cells. Single cell imaging revealed particles accumulation in the soma, but not in the nucleus. It can be seen that the cells became fluorescent with a dark shadow in the center reflecting the nuclei location (Fig. 2). For a quantitative assessment of MNPs internalization and the examination of the extent of cell penetration by these particles, PC12 cells were incubated with the nanoparticles for up to 72 h and the intracellular fluorescence intensity was measured by Flow cytometry. Figure 3a and b show the uptake of the MNPs by the cells, as a function of the duration of incubation, at 37 °C. After 1 h of incubation, MNPs can be detected in the cells. The fluorescence intensity increases as a linear function of incubation time, reaching a plateau after 24 h (Additional file 3: Figure S3). In a separate experiment we examined the effect of concentration of nanoparticles on cell uptake. PC12 cells were incubated with different concentrations of MNPs (0.01–0.5 mg/ml). The fluorescence intensity increased relatively to the MNPs concentration (Fig. 3c, d). These results demonstrate that cellular uptake of MNPs depends on the time of incubation and concentration of MNPs in the medium. These outcomes of cellular uptake are in good agreement with previous reported studies [34, 39]. An optimization of cell loading is necessary for various applications. For magnetic targeting and specifically for affecting cellular motility, a high amount of internalized iron oxide particles is desired. Using the uncoated-maghemite MNPs allows uploading high doses without reaching toxic levels. As iron-oxide particles also serve as contrast agents in magnetic resonance imaging (MRI) to label cells in vivo, uptake capacity is also critical to achieve significant signals [40, 41].

**Fig. 4:**
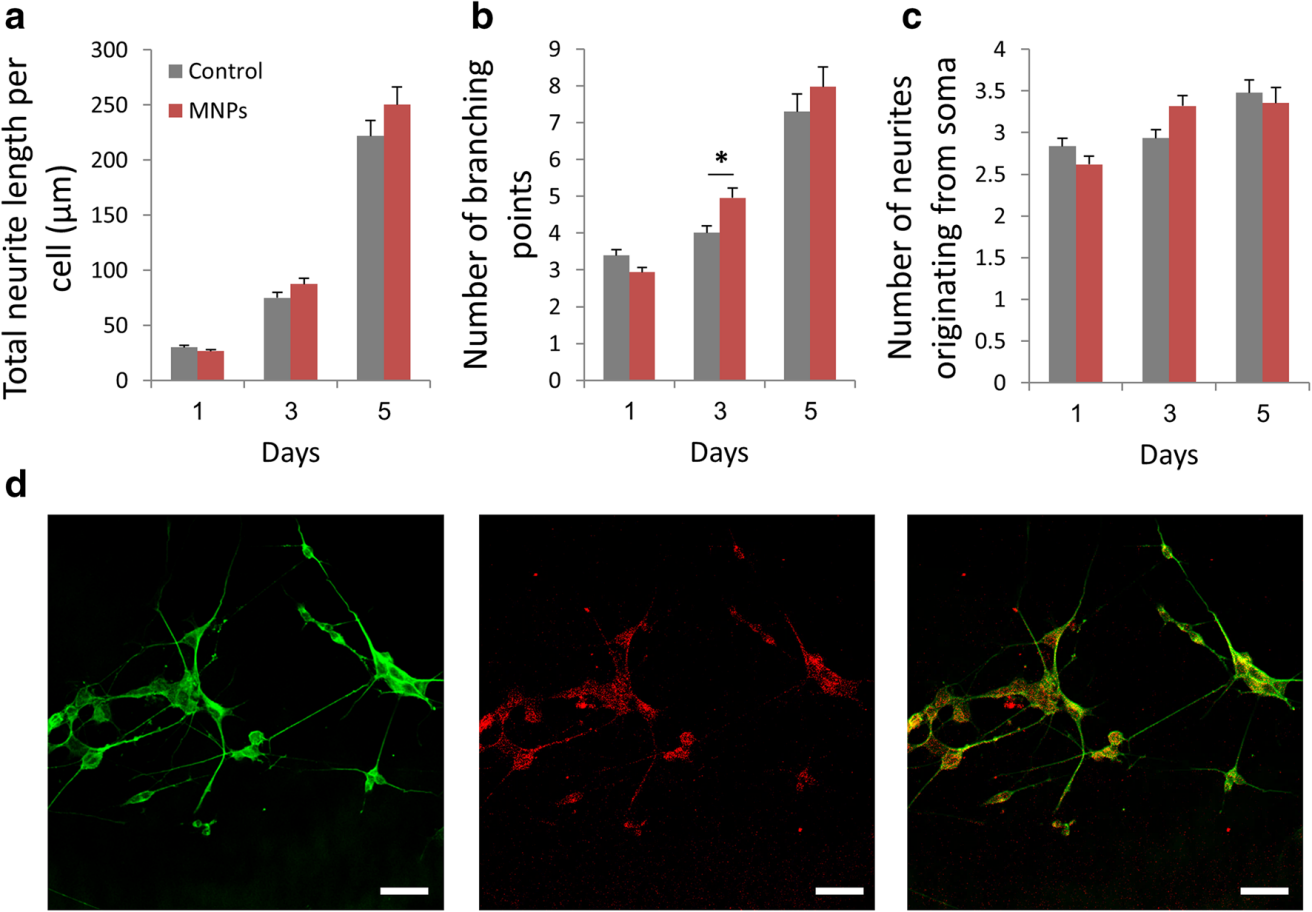
Morphological analysis of PC12 cells incubated with uncoated-maghemite MNPs (0.25 mg/ml) 1, 3 and 5 days after NGF treatment; **a** Total neurite length per cell; **b** Number of branching points; **c** Number of neurites originating from soma. T test, *p < 0.05.** d** Fluorescent confocal images of MNP-loaded cells after 5 days of NGF treatment; *Left* *image*: α-tubulin immunostaining of cells. *Middle image*: fluorescent MNPs uptaken by the cells. *Right image*: merged image.* Scale bar* = 50 µm

Additionally, we examined whether MNPs treatment interfered with PC12 cells normal differentiation capacity. We found that the MNPs-treated cells retained their ability to differentiate. The cells demonstrated neurite outgrowth and formation of a complex neuronal network (Fig. 4d). For a quantitative assessment of the differentiation process, morphological differentiation properties of MNPs-treated cells were measured at the single cell level (Fig. 4a–c). We measured the total neurite length per cell, the number of branching points and the number of neurites originating from soma, in a time dependent manner. Populations of cells were analyzed between 1–5 days after differentiation induction. Figure 4a demonstrates the average total neurite length for the control and treated cells. It can be seen that cells treated with MNPs developed similar neurites length to neurons in the control cultures. After 1 day of NGF treatment, we observed no significant difference in the number of branching points and number of neurites originating from soma in cells treated with MNPs in comparison to cells without MNPs treatment (Fig. 4b, c). Measurements of these parameters after 3 and 5 days in culture demonstrated similar trend, concluding that the uptake of the MNPs did not affect cellular differentiation potential.

**Fig. 5:**
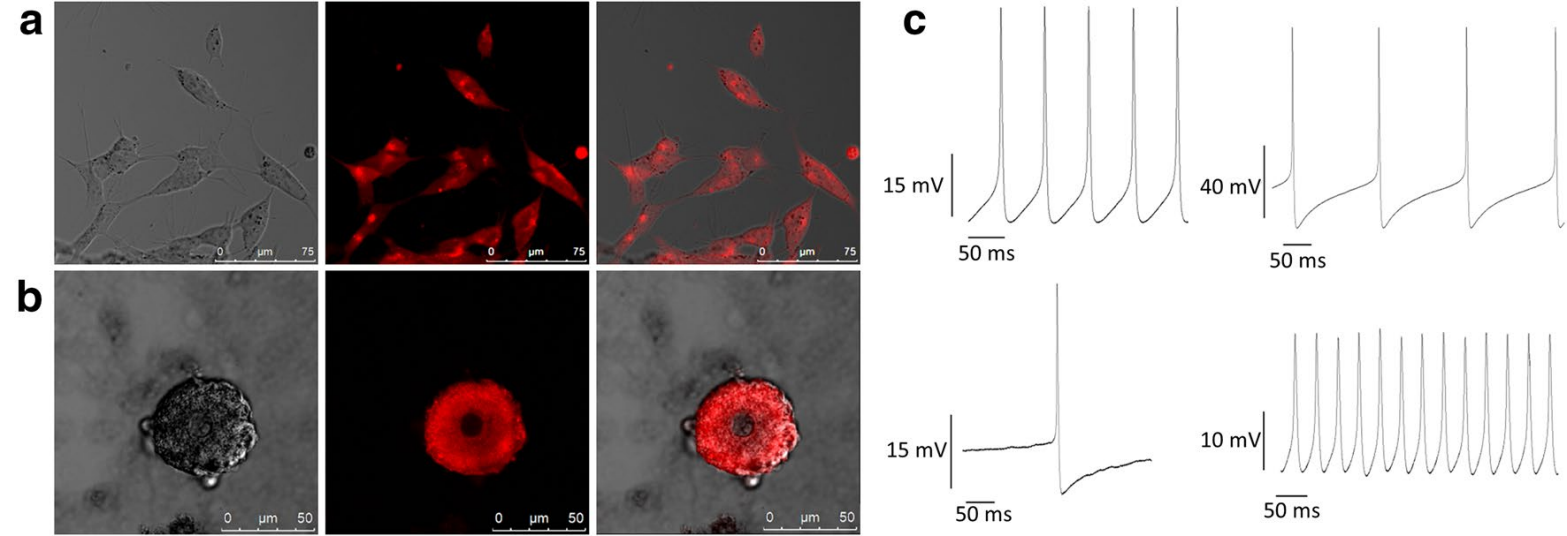
**a** Confocal microscopy images of SH-SY5Y cells at a single focal plane after 24 h of incubation with uncoated-maghemite MNPs. MNPs labeled with rhodamine (*red*) enter the cells; **b** Confocal microscopy images of primary leech neuron at a single focal plane after 24 h of incubation with the fluorescent MNPs. **c** Electrophysiological measurements of primary leech neurons in culture

Internalization of the maghemite nanoparticles into cells was also demonstrated within SH-SY5Y cells (Fig. 5a) and primary leech neurons (Fig. 5b). Leech neurons that have uptaken MNPs retained their regeneration capabilities as well. Electrophysiology measurements of isolated leech neurons, incubated with MNPs, revealed typical electrical activity and exhibited action potentials (Fig. 5c).

### Magnetic positioning of cells

**Fig. 6:**
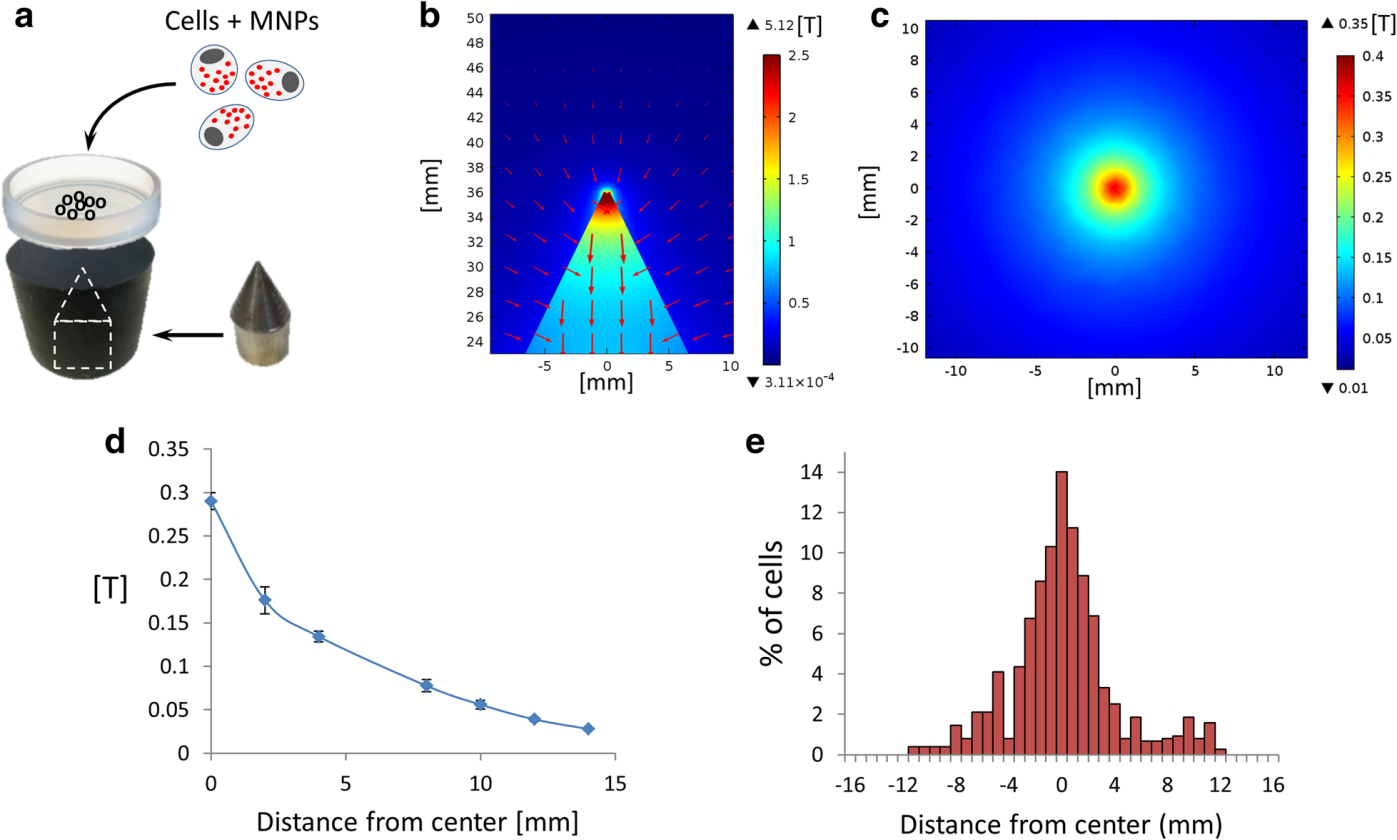
Magnetic positioning of PC12 cells. **a** Schematic illustration of the magnetic manipulation. Cells were incubated with MNPs and seeded on a plate placed above a Hiperco 50A magnetic tip. **b** Simulation of magnetic field lines and intensity in Comsol software. The image presents a* side view* of magnetic flux density of tip. *Red arrows* represent field direction, intensity is color coded (low intensity in *dark blue*, high intensity in *red*). **c** *Top view* simulation of magnetic flux density 0.9 mm above magnetic tip (thickness of plastic plate culture). **d** Actual measurements of magnetic field produced by single tip. *Error bars* represent standard deviation (*n* = 3). **e** *Graph* representing the cell distribution throughout the plate

**Fig. 7:**
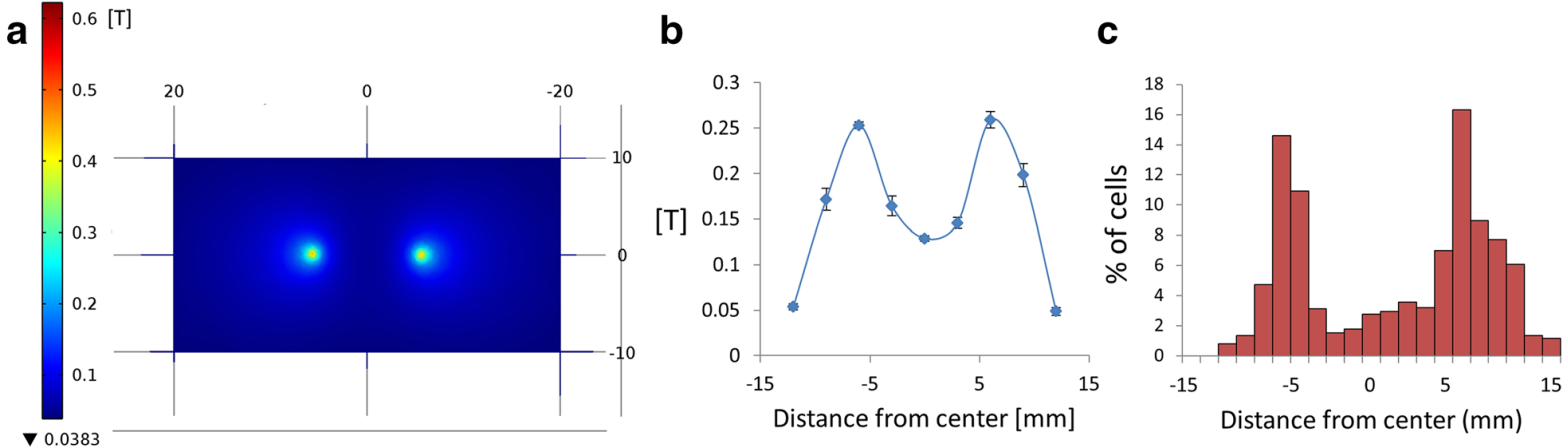
Positioning of PC12 cells using two magnetic tips. **a** Simulation of magnetic field intensity in Comsol software. **b** Actual measurements of magnetic field produced by two tips. *Error bars* represent standard deviation (*n* = 3). **c** Distribution of the cells throughout the plate culture

To demonstrate the ability to control cell motility and network organization by magnetic forces, the MNPs-loaded cells were placed in a culture dish and external magnetic fields of different profiles were applied. For schematic description of the experimental assay see Fig. 6a. First, local positioning of the MNP-loaded cells was achieved by using a single magnetic tip that was placed below the culture dish. A single magnetic tip produces a strong gradient of magnetic flux density, and the direction of the gradient is orientated towards the tip, as can be seen in the simulation results (Fig. 6b). Simulation of the magnetic field shows a maximal intensity value of about 0.3 T at the center of the plate, right above the magnetic tip, with a decrease in the magnetic field when moving away from the center. Actual measurements of the magnetic field produced by the magnetic tip show a similar trend (Fig. 6d). PC12 cells were incubated with MNPs overnight, washed twice and seeded on a 35 mm plate culture. A magnetic tip made of NdFeB N50 with 0.5 mm diameter was placed below the plate culture. The system was placed untouched for 3 days to allow the cells to migrate and organize under the influence of the magnetic field. Then, the entire culture dish was scanned and the distribution of cells was mapped. MNPs-loaded cells were found to be concentrated in the center of the dish, around the tip, and consequently ~70 % cell density was found within 3 mm from the center (Fig. 6a; Additional file 4: S4.A). Next, we used two magnet tips below the culture dish and placed them 1.2 cm apart. Actual measurements of the magnetic field matching the simulations demonstrating two high intensity peaks can be seen in Fig. 7a, b. This arrangement has changed the magnetic flux profile and led to different migration pattern. Figure 7c shows a clear two-peak distribution of the MNPs-loaded cells centered above the two magnetic sites (a representing culture can be seen in Additional file 4: Figure S4.B). These results demonstrate a correlation between the magnetic field forces and the cells distribution on the dish, enabling the use of this method as a tool for designing cellular organization, based on theoretical predictions.

Magnetic forces via MNPs allow a remote control of cell populations and may contribute to the rapid developing field of cell therapy. Our results indicate that magnetic cell positioning is feasible with this degree of cellular loading, and suggests that these particles may be suitable for potential cell targeting in vivo as well.

### Guiding neuronal growth by an external magnetic field

**Fig. 8:**
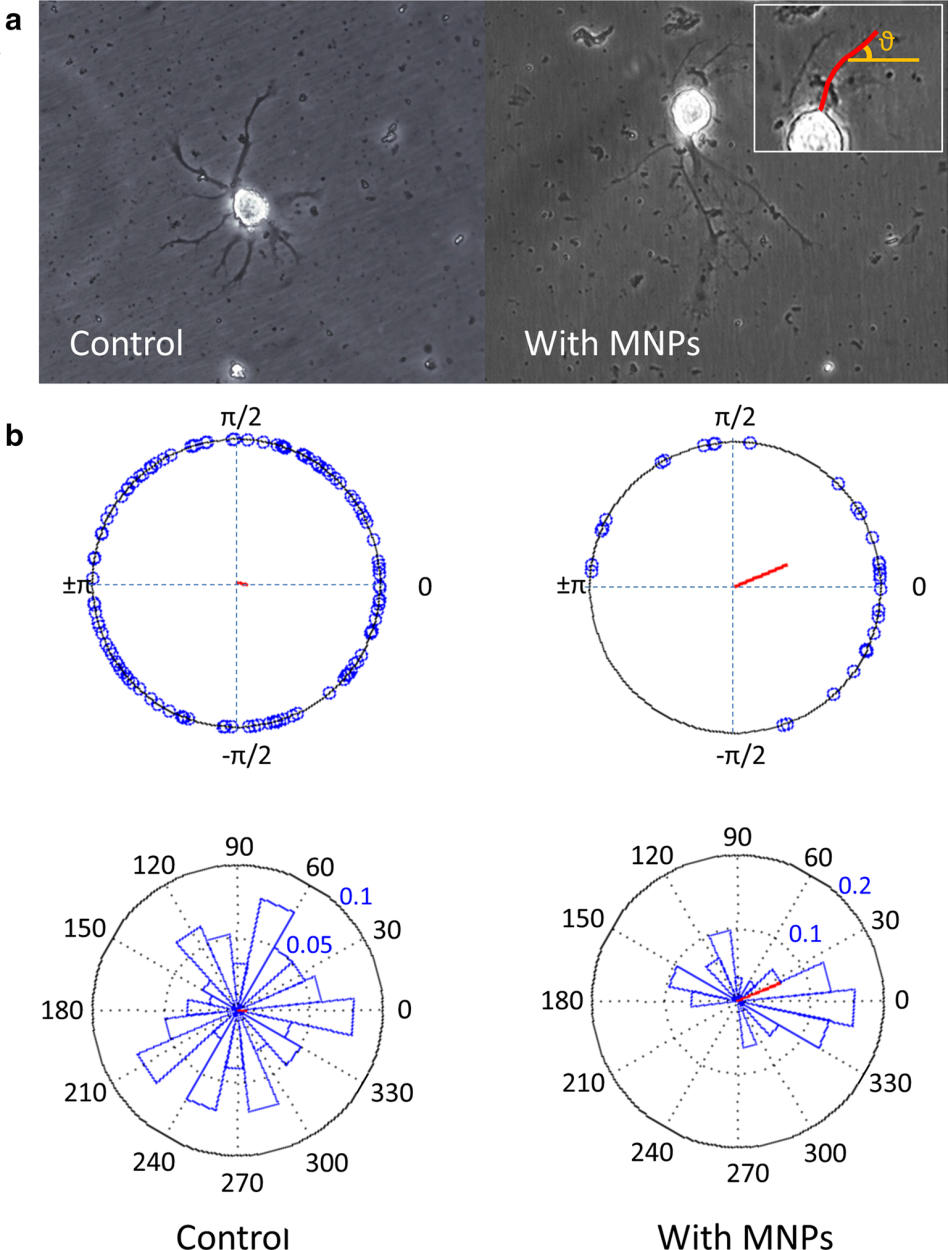
**a** Phase contrast images of leech neurons in culture in the presence of a magnetic field, either with MNPs or without (control). The neurites were traced and the direction of their tips was measured by calculating their angle relative to the direction of the magnetic field (inset). **b** Analysis of the direction of neurites outgrowth in the absence (control) or in the presence of MNPs, under an external magnetic field. The resultant vector length (marked in *red*) in the control group is 0.074 and the resultant vector length in the experiment group is 0.386 (p = 0.116, n = 312 and p = 0.009, n = 29, respectively, according to Rayleigh test for uniformity)

To examine the ability of magnetic forces to affect pattern of neurite growth, in addition to cell positioning, we have grown primary leech neurons loaded with MNPs under the influence of external magnetic field. Neurons were dissociated and incubated in medium enriched with MNPs and plated. Following cell adherence, an external magnetic field was applied, inducing a magnetic gradient during the growth process. We followed the growth and specifically the geometry of the neurons along the regeneration process and found that the magnetic field affected the neuronal outgrowth orientation. Figure 8a (left panel) demonstrates a single neuron that has regenerated spontaneously in culture, with no magnetic nanoparticles. It can be seen that neurites emerged out of the soma and developed towards all directions with no preference orientation. In contrast, in Fig. 8a (right panel) it can be seen that the neurites developed with a preferable direction, towards the magnetic gradient induced from the constant magnet that was placed on the right in this case. The distribution of angles between the magnetic field direction and the neurite tips (see inset of Fig. 8a for definition) is summarized in Fig. 8b. It can be seen that the distribution of neurite growth directions for the control neurons is homogeneous with no preferable direction. In contrast, neurons which have grown in the presence of MNPs under a magnetic field have developed neurites mostly towards the magnet direction (considered as direction 0º) and within a narrow range of angles (±30º). Few neurites have been developed towards the opposite direction (towards 180º), probably as a mechanical support for the cell [42].

## Conclusions

The ability to control neuronal migration and growth has important potential in therapeutics and neural regeneration studies. Applying mechanical forces via magnetic mediators has been presented as a useful approach in several biomedical applications. In this work we examined the interactions of different iron oxide nanoparticles with cells. We have focused on PC12 cells and shown that cell uptake is highly sensitive to the MNP type and incubation conditions highlighting the need for optimizing the MNP agents for specific application. The use of uncoated maghemite MNPs has led to maximal cellular penetration suggesting these particles as efficient candidates for magnetic-based neuronal manipulations. We have demonstrated a method to control the cellular distribution by magnetic field gradients based on theoretical considerations. Additionally, we used the MNPs to control neurite growth orientation of primary neurons along the process of neural network formation. Our results present the potential of MNPs as mediators for remote control of cells, as a novel therapeutic platform post neuronal injury.

## Additional files

10.1186/s12951-016-0190-0 Schematic illustration of magnetic tips.
10.1186/s12951-016-0190-0 XTT cell viability assay of PC12 cells incubated with uncoated-maghemite nanoparticles (0.6 mg/ml) for 1, 3 and 5 days.
10.1186/s12951-016-0190-0 Flow cytometry analysis of MNPs uptake by PC12 cells for up to 72 h of incubation.
10.1186/s12951-016-0190-0 Light microscopy images of cellular distribution under external magnetic fields produced by the single-tip and two-tips setups.

## References

[1] Nayak MS, Kim Y-S, Goldman M, Keirstead HS, Kerr DA (2006). Cellular therapies in motor neuron diseases. Biochim Biophys Acta.

[2] Cores J, Caranasos TG, Cheng K (2015). Magnetically targeted stem cell delivery for regenerative medicine. J Funct Biomater.

[3] Kilgus C, Heidsieck A, Ottersbach A, Roell W, Trueck C, Fleischmann BK, Gleich B, Sasse P (2012). Local gene targeting and cell positioning using magnetic nanoparticles and magnetic tips: comparison of mathematical simulations with experiments. Pharm Res.

[4] Slaughter GE, Bieberich E, Wnek GE, Wynne KJ, Guiseppi-Elie A (2004). Improving neuron-to-electrode surface attachment via alkanethiol self-assembly: an alternating current impedance study. Langmuir.

[5] Jungblut M, Knoll W, Thielemann C, Pottek M (2009). Triangular neuronal networks on microelectrode arrays: an approach to improve the properties of low-density networks for extracellular recording. Biomed Microdevices.

[6] Maher MP, Pine J, Wright J, Tai YC (1999). The neurochip: a new multielectrode device for stimulating and recording from cultured neurons. J Neurosci Methods.

[7] Hai A, Dormann A, Shappir J, Yitzchaik S, Bartic C, Borghs G, Langedijk JPM, Spira ME (2009). Spine-shaped gold protrusions improve the adherence and electrical coupling of neurons with the surface of micro-electronic devices. J R Soc Interface.

[8] Hanson L, Lin ZC, Xie C, Cui Y, Cui B (2012). Characterization of the cell-nanopillar interface by transmission electron microscopy. Nano Lett.

[9] Sensenig R, Sapir Y, MacDonald C, Cohen S, Polyak B (2012). Magnetic nanoparticle-based approaches to locally target therapy and enhance tissue regeneration in vivo. Nanomedicine (Lond).

[10] Krishnan KM (2010). Biomedical nanomagnetics: a spin through possibilities in imaging, diagnostics, and therapy. IEEE Trans Magn.

[11] Pankhurst QA, Connolly J, Jones SK, Dobson J (2003). Applications of magnetic nanoparticles in biomedicine. J Phys D Appl Phys.

[12] Polak P, Shefi O (2015). Nanometric agents in the service of neuroscience: manipulation of neuronal growth and activity using nanoparticles. Nanomedicine.

[13] Marcus M, Skaat H, Alon N, Margel S, Shefi O (2014). NGF-conjugated iron oxide nanoparticles promote differentiation and outgrowth of PC12 cells. Nanoscale.

[14] Skaat H, Ziv-Polat O, Shahar A, Margel S (2011). Enhancement of the growth and differentiation of nasal olfactory mucosa cells by the conjugation of growth factors to functional nanoparticles. Bioconjug Chem.

[15] Ziv-Polat O, Shahar A, Levy I, Skaat H, Neuman S, Fregnan F, Geuna S, Grothe C, Haastert-Talini K, Margel S (2014). The role of neurotrophic factors conjugated to iron oxide nanoparticles in peripheral nerve regeneration: in vitro studies. Biomed Res Int.

[16] Yanai A, Häfeli UO, Metcalfe AL, Soema P, Addo L, Gregory-Evans CY, Po K, Shan X, Moritz OL, Gregory-Evans K (2012). Focused magnetic stem cell targeting to the retina using superparamagnetic iron oxide nanoparticles. Cell Transplant.

[17] Carenza E, Barceló V, Morancho A, Levander L, Boada C, Laromaine A, Roig A, Montaner J, Rosell A (2014). In vitro angiogenic performance and in vivo brain targeting of magnetized endothelial progenitor cells for neurorepair therapies. Nanomedicine.

[18] Nishida K, Tanaka N, Nakanishi K, Kamei N, Hamasaki T, Yanada S, Mochizuki Y, Ochi M (2006). Magnetic targeting of bone marrow stromal cells into spinal cord: through cerebrospinal fluid. NeuroReport.

[19] Polyak B, Fishbein I, Chorny M, Alferiev I, Williams D, Yellen B, Friedman G, Levy RJ (2008). High field gradient targeting of magnetic nanoparticle-loaded endothelial cells to the surfaces of steel stents. Proc Natl Acad Sci USA.

[20] Ranney DF, Huffaker HH (1987). Magnetic microspheres for the targeted controlled release of drugs and diagnostic agents. Ann N Y Acad Sci.

[21] Busquets M, Espargaró A, Sabaté R, Estelrich J (2015). Magnetic nanoparticles cross the blood-brain barrier: when physics rises to a challenge. Nanomaterials.

[22] Jain S, Mishra V, Singh P, Dubey P, Saraf D, Vyas S (2003). RGD-anchored magnetic liposomes for monocytes/neutrophils-mediated brain targeting. Int J Pharm.

[23] Riegler J, Wells JA, Kyrtatos PG, Price AN, Pankhurst QA, Lythgoe MF (2010). Targeted magnetic delivery and tracking of cells using a magnetic resonance imaging system. Biomaterials.

[24] Lee CS, Lee H, Westervelt RM (2001). Microelectromagnets for the control of magnetic nanoparticles. Appl Phys Lett.

[25] Tseng P, Judy JW, Di Carlo D (2012). Magnetic nanoparticle-mediated massively parallel mechanical modulation of single-cell behavior. Nat Methods.

[26] Lee H, Liu Y, Ham D, Westervelt RM (2007). Integrated cell manipulation system—CMOS/microfluidic hybrid. Lab Chip.

[27] Alon N, Havdala T, Skaat H, Baranes K, Marcus M, Levy I, Margel S, Sharoni A, Shefi O (2015). Magnetic micro-device for manipulating PC12 cell migration and organization. Lab Chip.

[28] Tseng P, Di Carlo D, Judy JW (2009). Rapid and dynamic intracellular patterning of cell-internalized magnetic fluorescent nanoparticles. Nano Lett.

[29] Baranes K, Chejanovsky N, Alon N, Sharoni A, Shefi O (2012). Topographic cues of nano-scale height direct neuronal growth pattern. Biotechnol Bioeng.

[30] Baranes K, Shevach M, Shefi O, Dvir T (2015). Gold nanoparticle-decorated scaffolds promote neuronal differentiation and maturation. Nano Lett.

[31] Margel S, Gura S. Nucleation and growth of magnetic metal oxide nanoparticles and its use. 2006: Israel Patent No. WO9962079.

[32] Meijering E, Jacob M, Sarria J-CF, Steiner P, Hirling H, Unser M (2004). Design and validation of a tool for neurite tracing and analysis in fluorescence microscopy images. Cytometry A.

[33] Foged C, Brodin B, Frokjaer S, Sundblad A (2005). Particle size and surface charge affect particle uptake by human dendritic cells in an in vitro model. Int J Pharm.

[34] Thorek DLJ, Tsourkas A (2008). Size, charge and concentration dependent uptake of iron oxide particles by non-phagocytic cells. Biomaterials.

[35] Connell JJ, Patrick PS, Yu Y, Lythgoe MF, Kalber TL (2015). Advanced cell therapies: targeting, tracking and actuation of cells with magnetic particles. Regen Med.

[36] Mahmoudi M, Hofmann H, Rothen-Rutishauser B, Petri-Fink A (2012). Assessing the in vitro and in vivo toxicity of superparamagnetic iron oxide nanoparticles. Chem Rev.

[37] Pisanic TR, Blackwell JD, Shubayev VI, Fiñones RR, Jin S (2007). Nanotoxicity of iron oxide nanoparticle internalization in growing neurons. Biomaterials.

[38] Kim JA, Lee N, Kim BH, Rhee WJ, Yoon S, Hyeon T, Park TH (2011). Enhancement of neurite outgrowth in PC12 cells by iron oxide nanoparticles. Biomaterials.

[39] Schlorf T, Meincke M, Kossel E, Glüer C-C, Jansen O, Mentlein R (2010). Biological properties of iron oxide nanoparticles for cellular and molecular magnetic resonance imaging. Int J Mol Sci.

[40] Taylor A, Herrmann A, Moss D, Sée V, Davies K, Williams SR, Murray P (2014). Assessing the efficacy of nano- and micro-sized magnetic particles as contrast agents for MRI cell tracking. PLoS One.

[41] Meng X, Seton HC, Lu LT, Prior IA, Thanh NTK, Song B (2011). Magnetic CoPt nanoparticles as MRI contrast agent for transplanted neural stem cells detection. Nanoscale.

[42] Shefi O, Harel A, Chklovskii DB, Ben-Jacob E, Ayali A (2004). Biophysical constraints on neuronal branching. Neurocomputing.

