# Supplementary figures and images for: Iron oxide nanoparticles for neuronal cell applications: uptake study and magnetic manipulations
Source: J Nanobiotechnology. 2016 May 14;14:37. doi: 10.1186/s12951-016-0190-0 (PMC4867999; doi:10.1186/s12951-016-0190-0)

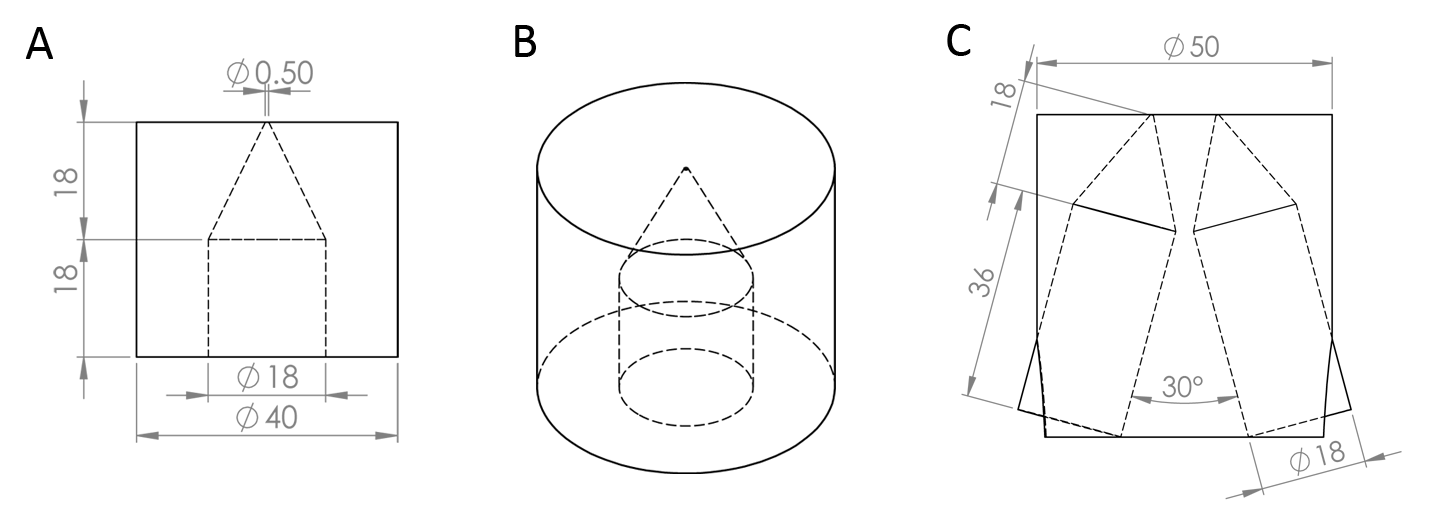

Supplement: Supplementary file 1 — 10.1186/s12951-016-0190-0 Schematic illustration of magnetic tips. [file 12951_2016_190_MOESM1_ESM.tif]

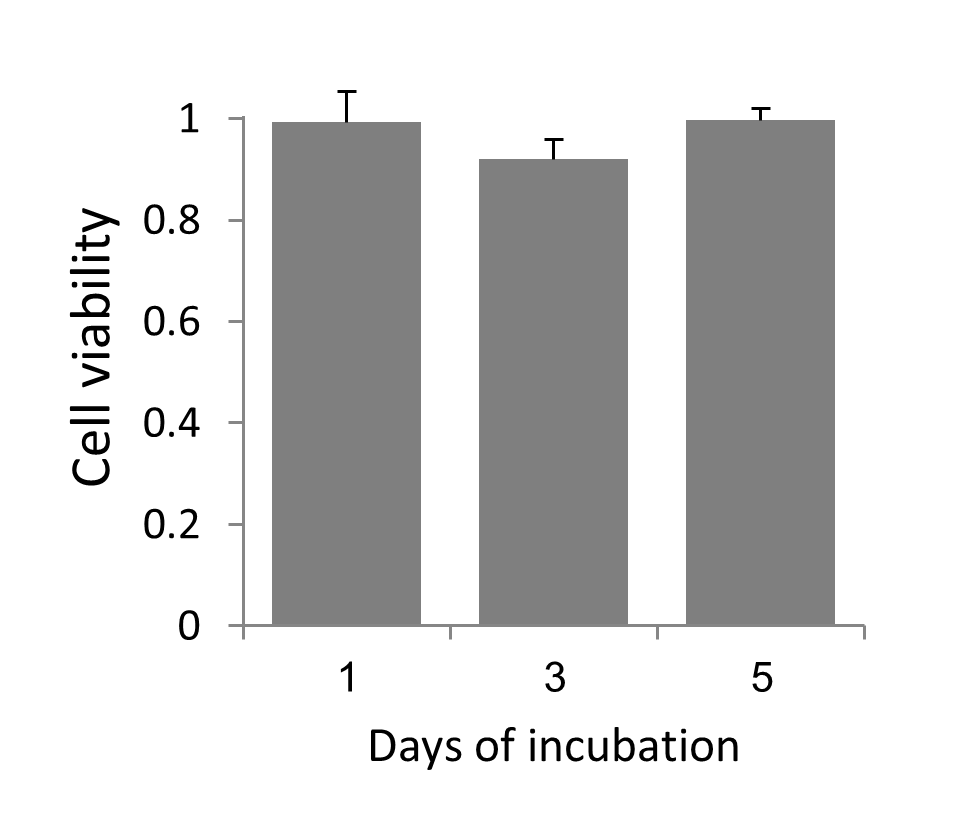

Supplement: Supplementary file 2 — 10.1186/s12951-016-0190-0 XTT cell viability assay of PC12 cells incubated with uncoated-maghemite nanoparticles (0.6 mg/ml) for 1, 3 and 5 days. [file 12951_2016_190_MOESM2_ESM.tif]

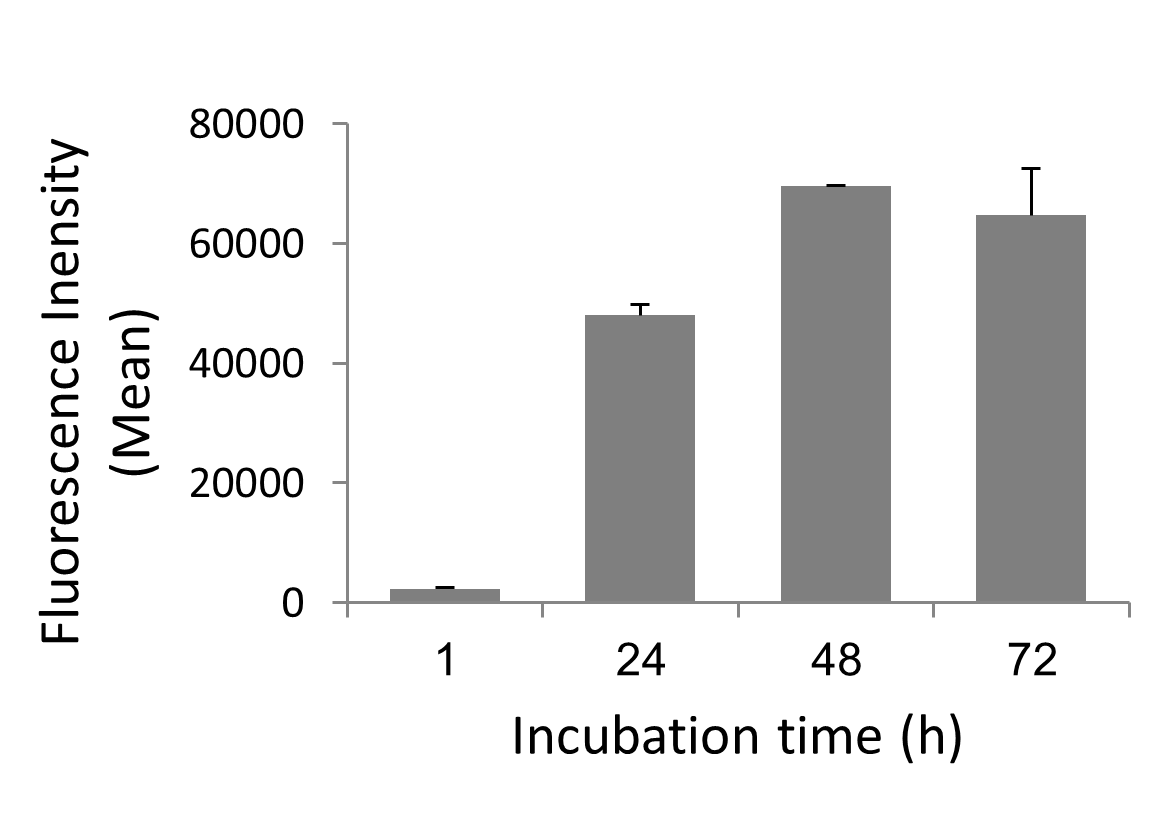

Supplement: Supplementary file 3 — 10.1186/s12951-016-0190-0 Flow cytometry analysis of MNPs uptake by PC12 cells for up to 72 h of incubation. [file 12951_2016_190_MOESM3_ESM.tif]

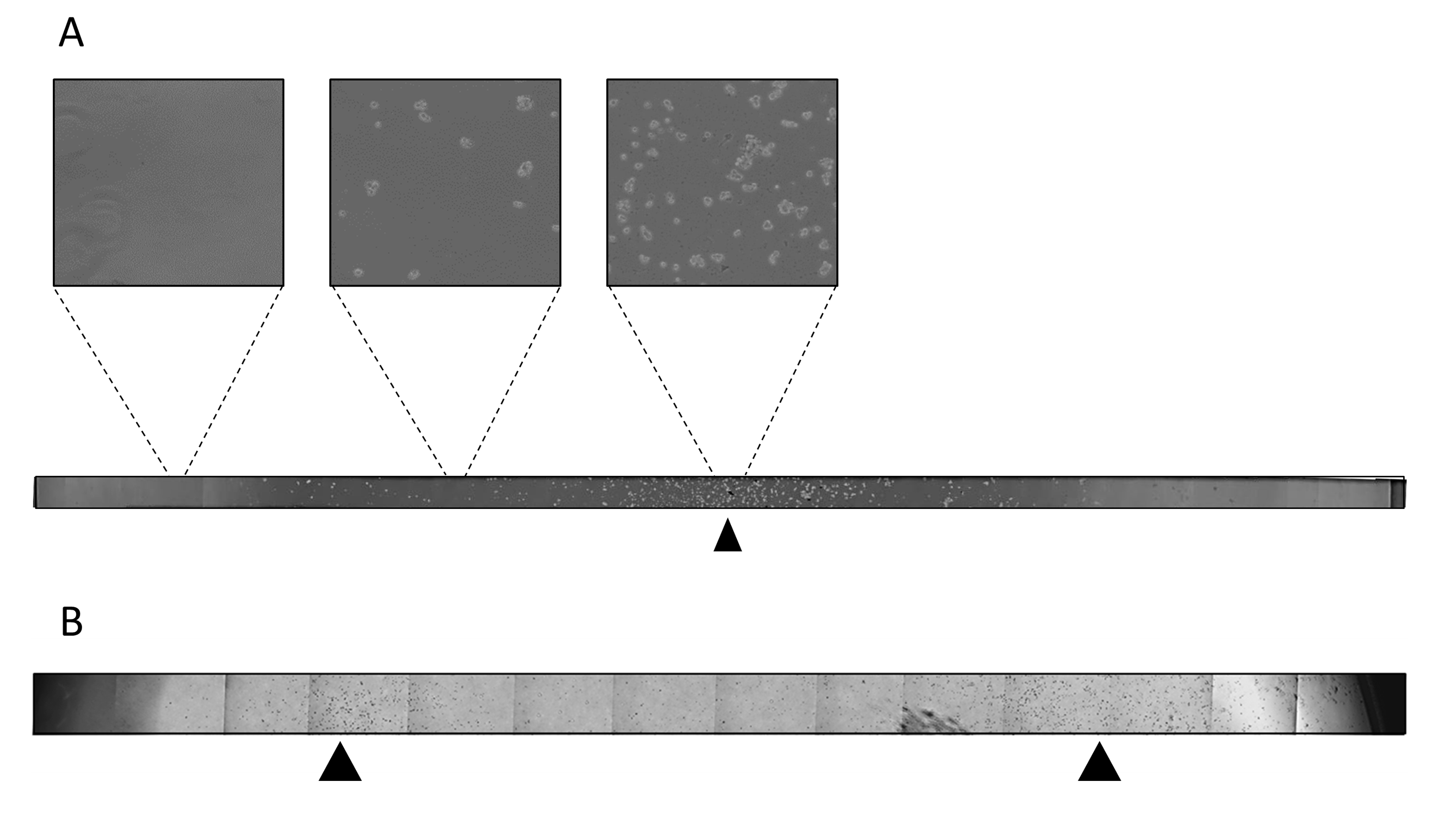

Supplement: Supplementary file 4 — 10.1186/s12951-016-0190-0 Light microscopy images of cellular distribution under external magnetic fields produced by the single-tip and two-tips setups. [file 12951_2016_190_MOESM4_ESM.tif]
